# Supplementary material for: Major satellite repeat RNA stabilize heterochromatin retention of Suv39h enzymes by RNA-nucleosome association and RNA:DNA hybrid formation
Source: eLife. 2017 Aug 1;6:e25293. doi: 10.7554/eLife.25293 (PMC5538826; doi:10.7554/eLife.25293)
Supplement: Supplementary file 1. — Additional sequences of RNA oligonucleotides (minor satellite repeats, LINE L1 MdA, SINE B1, pRNA and TERRA) used for EMSA are indicated in Figure 3—figure supplement 1. DOI: http://dx.doi.org/10.7554/eLife.25293.018 [file elife-25293-supp1.docx]

**Supplemental Table 1** Oligonucleotide sequences used in this study

­­

| Target | Sequence (5’ -> 3’) | Use |
| --- | --- | --- |
| Suv39h2-FL- F | 5’-ATGGCGGCGGCCAGGGC | Suv39h2 cloning |
| Suv39h2-FL- R | 5’-TCAGTTGAGGTAACCTCTGCAAG | Suv39h2 cloning |
| MajSat - F | 5’-TGGAATATGGCGAGAAAACTG | RT-qPCR, ChIP, RDIP |
| MajSat - R | 5’-AGGTCCTTCAGTGGGCATTT | RT-qPCR, ChIP, RDIP |
| MajSat-Subrep2-Cy5-RNA - F | **Cy5**-5’-GAAAUAUGGCGAGGAAAACUGAAAAAGGUGGAAAA | EMSA |
| MajSat-Subrep2-Cy5-RNA - R | **Cy5**-5’-UUUUCCACCUUUUUCAGUUUUCCUCGCCAUAUUUC | EMSA |
| MajSat-Subrep2-Cy5-DNA - F | **Cy5**-5’-GAAATATGGCGAGGAAAACTGAAAAAGGTGGAAAA | EMSA, RNaseA/H activity assay |
| MajSat-Subrep2-Cy5-DNA - R | **Cy5**-5’-TTTTCCACCTTTTTCAGTTTTCCTCGCCATATTTC | EMSA, RNaseA/H activity assay |
| MajSat-Subrep2-Cy3-RNA - F | **Cy3**-5’-GAAAUAUGGCGAGGAAAACUGAAAAAGGUGGAAAA | RNaseA/H activity assay |
| MajSat-Subrep2-Cy3-RNA - R | **Cy3**-5’-UUUUCCACCUUUUUCAGUUUUCCUCGCCAUAUUUC | RNaseA/H activity assay |
| MajSat-Subrep2-RNA - F | 5’-GAAAUAUGGCGAGGAAAACUGAAAAAGGUGGAAAA | S9.6 Ab characterization |
| MajSat-Subrep2-RNA - R | 5’-UUUUCCACCUUUUUCAGUUUUCCUCGCCAUAUUUC | S9.6 Ab characterization |
| MajSat-Subrep2-DNA - F | 5’-GAAATATGGCGAGGAAAACTGAAAAAGGTGGAAAA | S9.6 Ab characterization |
| MajSat-Subrep2-DNA - R | 5’-TTTTCCACCTTTTTCAGTTTTCCTCGCCATATTTC | S9.6 Ab characterization |
| MajSat-Subrep4-DNA - F | 5’-ATGAGAAACATCCACTTGACGACTTGAAAAATGACGAAATCACT | Northern blot |
| MajSat-Subrep4-DNA - R | 5’-AGTGATTTCGTCATTTTTCAAGTCGTCAAGTGGATGTTTCTCAT | Northern blot |
| L1MdA - F | 5’-ACTGCGGTACATAGGGAAGC | RT-qPCR, RDIP |
| L1MdA - R | 5’-TGTGATCCACTCACCAGAGG | RT-qPCR, RDIP |
| L1MdA_Nor - F | 5’-GTACATAGGGAAGCAGGCTACCCGGGCCTGATCTGGGGCACAAG | Northern blot |
| L1MdA_Nor - R | 5’-CTTGTGCCCCAGATCAGGCCCGGGTAGCCTGCTTCCCTATGTAC | Northern blot |
| SINEB1 - F | 5’-GTGGCGCACGCCTTTAATC | RT-qPCR, RDIP |
| SINEB1 - R | 5’-GACAGGGTTTCTCTGTGTAG | RT-qPCR, RDIP |
| SINEB2 - F | 5’-GAGATGGCTCAGTGGTTAAG | RT-qPCR |
| SINEB2 - R | 5’-CTGTCTTCAGACACTCCAG | RT-qPCR |
| Gapdh - F | 5’-TGAACGGGAAGCTCACTGG | RT-qPCR |
| Gapdh - R | 5’-TCCACCACCCTGTTGCTGTA | RT-qPCR |
| Hprt - F | 5’-AGTGATAGATCCATTCCTATGACTGTAG | RT-qPCR |
| Hprt - R | 5’-GTTAAAGTTGAGAGATCATCTCCACC | RT-qPCR |
| Actin - F | 5’-AAGGCCAACCGTGAAAAGAT | RT-qPCR |
| Actin - R | 5’-GTGGTACGACCAGAGGCATAC | RT-qPCR |
| Tubulin - F | 5’-GACAGAGGCAAACTGAGCACC | RT-qPCR |
| Tubulin - R | 5’-CAACGTCAAGACGGCCGTGTG | RT-qPCR |
| 28S-rRNA - F | 5’-CCGCCCAGGCGGAACGATAC | RT-qPCR |
| 28S-rRNA - R | 5’-GCAGCGAGGGAGCTGCTCTG | RT-qPCR |
| pSAT-MSR-6FAM | **6FAM**-5’-TCCTGCAGGTTTAAACGAATTC | SHAPE |
| pSAT-MSR-VIC | **VIC**-5’-TCCTGCAGGTTTAAACGAATTC | SHAPE |
| pSAT-MSR-NED | **NED**-5’-TCCTGCAGGTTTAAACGAATTC | SHAPE |
| pSAT-MSR-PET | **PET**-5’-TCCTGCAGGTTTAAACGAATTC | SHAPE |
| pEX-L1MdA_F-6FAM | **6FAM**-5’-GTGATCCACTCACCAGAGGTCTT | SHAPE |
| pEX-L1MdA_F-VIC | **VIC**-5’-GTGATCCACTCACCAGAGGTCTT | SHAPE |
| pEX-L1MdA_F-NED | **NED**-5’-GTGATCCACTCACCAGAGGTCTT | SHAPE |
| pEX-L1MdA_F-PET | **PET**-5’-GTGATCCACTCACCAGAGGTCTT | SHAPE |
| pEX-L1MdA_R-6FAM | **6FAM**-5’-TGCCTACCCCAATCCAATCGC | SHAPE |
| pEX-L1MdA_R-VIC | **VIC**-5’-TGCCTACCCCAATCCAATCGC | SHAPE |
| pEX-L1MdA_R-NED | **NED**-5’-TGCCTACCCCAATCCAATCGC | SHAPE |
| pEX-L1MdA_R-PET | **PET**-5’-TGCCTACCCCAATCCAATCGC | SHAPE |

Additional sequences of the RNA oligonucleotides (minor satellite repeats, L1MdA 5’UTR, SINE B1, pRNA, TERRA and poly(A)) used for EMSA are indicated in Figure 3 - figure supplement 1.
